# Acute respiratory failure as a rare presentation of non-fibrotic hypersensitivity pneumonitis: Case series

**DOI:** 10.1097/MD.0000000000049011

**Published:** 2026-05-29

**Authors:** Katarzyna B. Lewandowska, Lucyna Opoka, Iwona Bartoszuk, Małgorzata E. Jędrych, Piotr Radwan-Rohrenschef, Kamila Deutsch, Adriana Roży, Lilia Jakubowska, Witold Z. Tomkowski, Małgorzata Sobiecka, Monika Szturmowicz

**Affiliations:** a 1st Department of Lung Diseases, National Tuberculosis and Lung Diseases Research Institute, Warsaw, Poland; b Department of Radiology, National Tuberculosis and Lung Diseases Research Institute, Warsaw, Poland; c Department of Genetics and Clinical Immunology, National Tuberculosis and Lung Diseases Research Institute, Warsaw, Poland.

**Keywords:** acute respiratory failure, case report, high-resolution computed tomography, hypersensitivity pneumonitis, therapy

## Abstract

**Rationale:**

Hypersensitivity pneumonitis (HP) is an increasingly recognized interstitial lung disease caused by exposure to inhaled, mostly organic, antigens. The non-fibrotic type of HP generally follows a benign course. We present 3 patients with a rare presentation of non-fibrotic HP—acute respiratory failure.

**Patient concerns:**

2 females and 1 male, presented to the pulmonary department due to resting dyspnea, cough and low-grade fever. Arterial blood gases revealed profound hypoxemia. No relevant abnormalities were found in the blood tests. In high-resolution computed tomography (HRCT), signs of extensive interstitial lung disease were present. Infection was excluded based on genetic testing and cultures. Pulmonary function tests revealed decrease in forced vital capacity (FVC) and transfer factor of the lungs for carbon monoxide (TLco).

**Diagnoses:**

Non-fibrotic HP was diagnosed based on positive exposure to organic antigens, characteristic lung HRCT pattern, and bronchoalveolar lavage lymphocytosis (in 2 cases).

**Interventions:**

Oxygen therapy was implemented through the nasal tube, accompanied by intravenous and subsequently oral steroids, gradually tapered.

**Outcomes:**

Rapid clinical improvement, near-complete regression of interstitial lung disease, significant increase in lung volumes and lung transfer factor for carbon monoxide were observed and maintained with low-dose therapy.

**Lessons:**

Non-fibrotic HP rarely presents as a life-threatening interstitial lung disease, requiring immediate immunosuppressive treatment. In some patients with extensive lung involvement, prolonged steroid therapy is necessary to achieve complete resolution of lung opacities and clinical improvement.

## 1. Introduction

Hypersensitivity pneumonitis (HP) is currently 1 of the 3 most common interstitial lung diseases (ILD),^[1]^ with an estimated incidence of 0.1–1.94 cases per 100,000 and a prevalence of 0.45–2.7 per 100,000.^[2–5]^ According to the United States (US) epidemiological data, HP prevalence is higher in women and in individuals over 65 years old.^[4]^ The ATS/JRS/ALAT guidelines published in 2020 distinguish 2 forms of HP: non-fibrotic (non-fHP) and fibrotic (fHP).^[6]^ Progressive lung fibrosis occurs in 58% of fHP patients and is associated with a significant decline in life expectancy.^[7,8]^ The emerging role of anti-fibrotic treatment in fHP has been discussed by many authors recently.^[9–11]^

Conversely, non-fHP is considered a milder form of the disease.^[12]^ Removing the inciting antigen from the environment is recommended as the primary intervention.^[12,13]^ However, rarely, patients diagnosed with non-fHP may present with an acute onset and extensive lung involvement, requiring urgent immunosuppressive treatment due to severe respiratory failure. The diagnostic and treatment challenges of such cases are discussed below.

## 2. Cases description

### 2.1. Case 1

**Figure 1.**
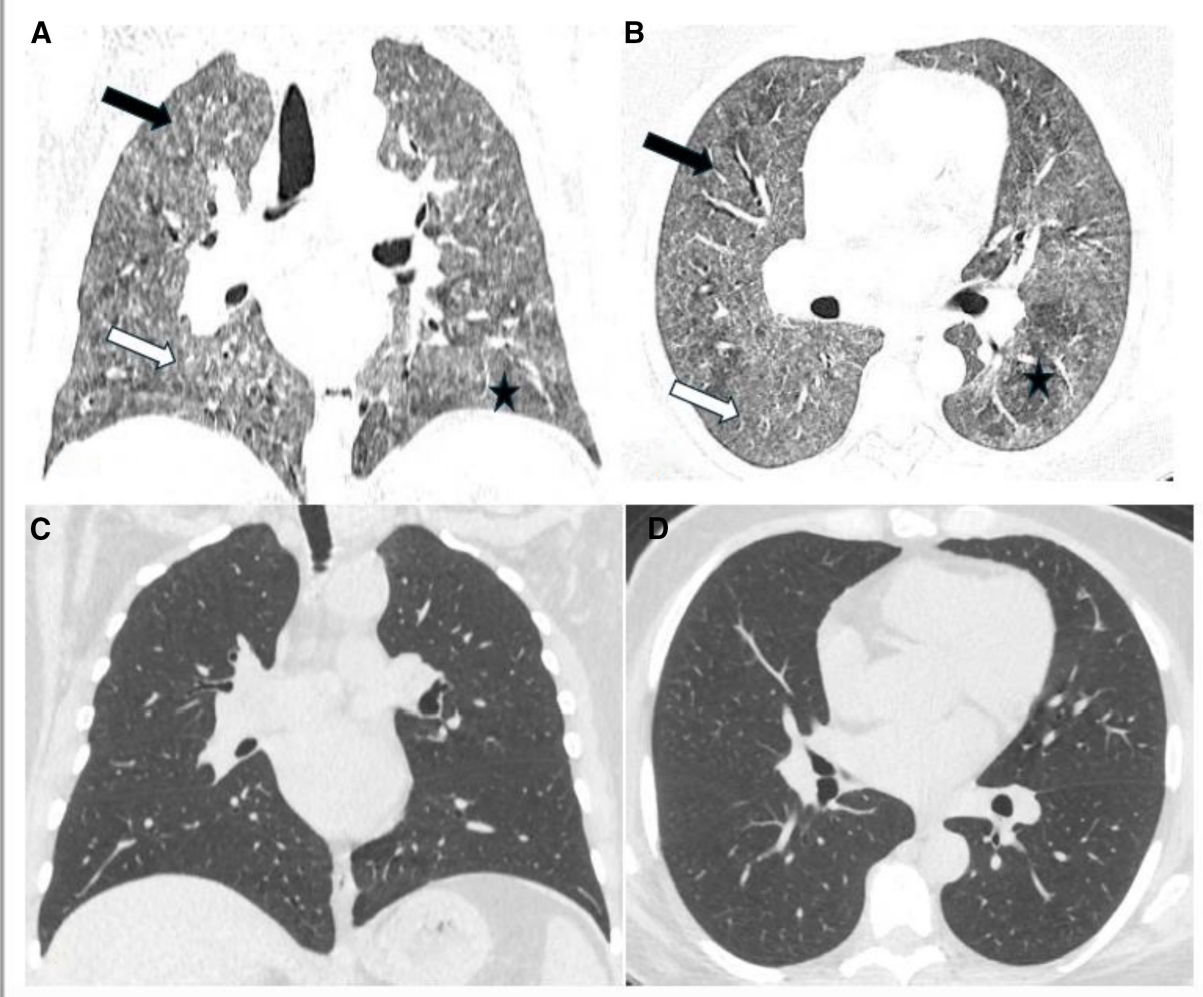
Patient 1 - High-resolution computed tomography (HRCT) scans, coronal and axial planes. (A, B) baseline scans, (C, D) follow-up scanes. (A, B) Bilateral diffuse ill-defined centrilobular nodules (black arrows), an increase in lung density with visibility of vessels and bronchial walls - ground glass opacities (white arrows) and air trapping indicating nonfibrous HP (asterisks); (C, D) nearly complete regression of ground glass opacities.

A 43-year-old obese woman, nonsmoker, with type 2 diabetes, arterial hypertension, and nodular goiter in the euthyroid stage, was admitted to the pulmonary unit in April 2023 due to acute hypoxemic respiratory failure. In March 2021, she was diagnosed with mild SARS-CoV-2 infection. Since the beginning of 2023, she has complained of a tiring, nonproductive cough, increasing limitations in exercise capacity, and occasional episodes of low-grade fever. She lived in her own house, heated with wood. She owned a cat and a rabbit; occasionally, she cared for her daughter parrot. On admission, she exhibited resting dyspnea, an oxygen saturation (SpO_2_) of 80%, and bilateral respiratory crackles over the lung bases. A chest radiograph revealed bilateral reticular lung infiltrates in the medial and lower parts of her lungs. A computed tomography (CT) scan of the chest showed diffuse bilateral centrilobular nodules and ground-glass opacities, with mosaic lung attenuation, suggestive of air trapping (figure 1A, B).

Respiratory infections were excluded, including nasopharyngeal smears for SARS-CoV-2 PCR, sputum culture, sputum *Pneumocystis jiroveci*i PCR, and urine antigen tests for *Legionella pneumophila*.

Based on the positive history of exposure to bird droppings and other organic antigens and the typical HRCT pattern, non-fHP was recognized.

Oxygen therapy with increasing flow up to 6 L/min through an intranasal catheter was applied, resulting in an increase of SpO_2_ to 95%. Intravenous methylprednisolone at 0.5 mg/kg/day (40 mg/day) was initiated. Oxygen flow was reduced from 6 L/min to 1 L/min over ten days of therapy. On fiber-optic bronchoscopy (FOB), performed on the 10th day of treatment, diffuse inflammatory lesions were observed in the bronchial mucosa. Cultures of respiratory specimens for bacteria were negative. Bronchoalveolar lavage (BAL) showed a total cell count of 27 × 10^^6^ (normal: <10 × 10^^6^), with lymphocytes at 51% (normal: <15%), neutrophils at 8% (normal: <3%), and eosinophils at 0.2% (normal: <0.5%). Steroid therapy was continued with prednisone at 30 mg/day, gradually tapered to 20 mg/day. After 4 months, follow-up HRCT demonstrated nearly complete regression of lung infiltrates (Figure 1C,D). Additionally, significant increases in lung volumes and transfer factor for carbon monoxide (TLco), along with a decrease in lung hyperinflation (RV/TLC), were noted (Table 1).

**Table 1:** Initial and post treatment pulmonary function tests results of the 3 presented patients.

	FVC (% pred.)		TLC (%pred.)		RV/TLC (%pred.)		TLco (% pred.)	
	Pre	Post	Pre	Post	Pre	Post	Pre	Post
Case 1	60	98	83	112	140	109	44	76
Case 2	80	92	92	101	116	106	60	81
Case 3	74	118	87	113	113	96	28	60

FVC = forced vital capacity, post = post treatment value, pre = initial value, RV = residual volume, TLC = total lung capacity, TLco = transfer factor of the lungs for carbon monoxide.

Currently, the patient is being treated with prednisone at a maintenance dose of 15 mg/day. She has no contact with animals or parrots. However, she still lives in the same house. No further disease exacerbations have been observed.

### 2.2. Case 2

**Figure 2.**
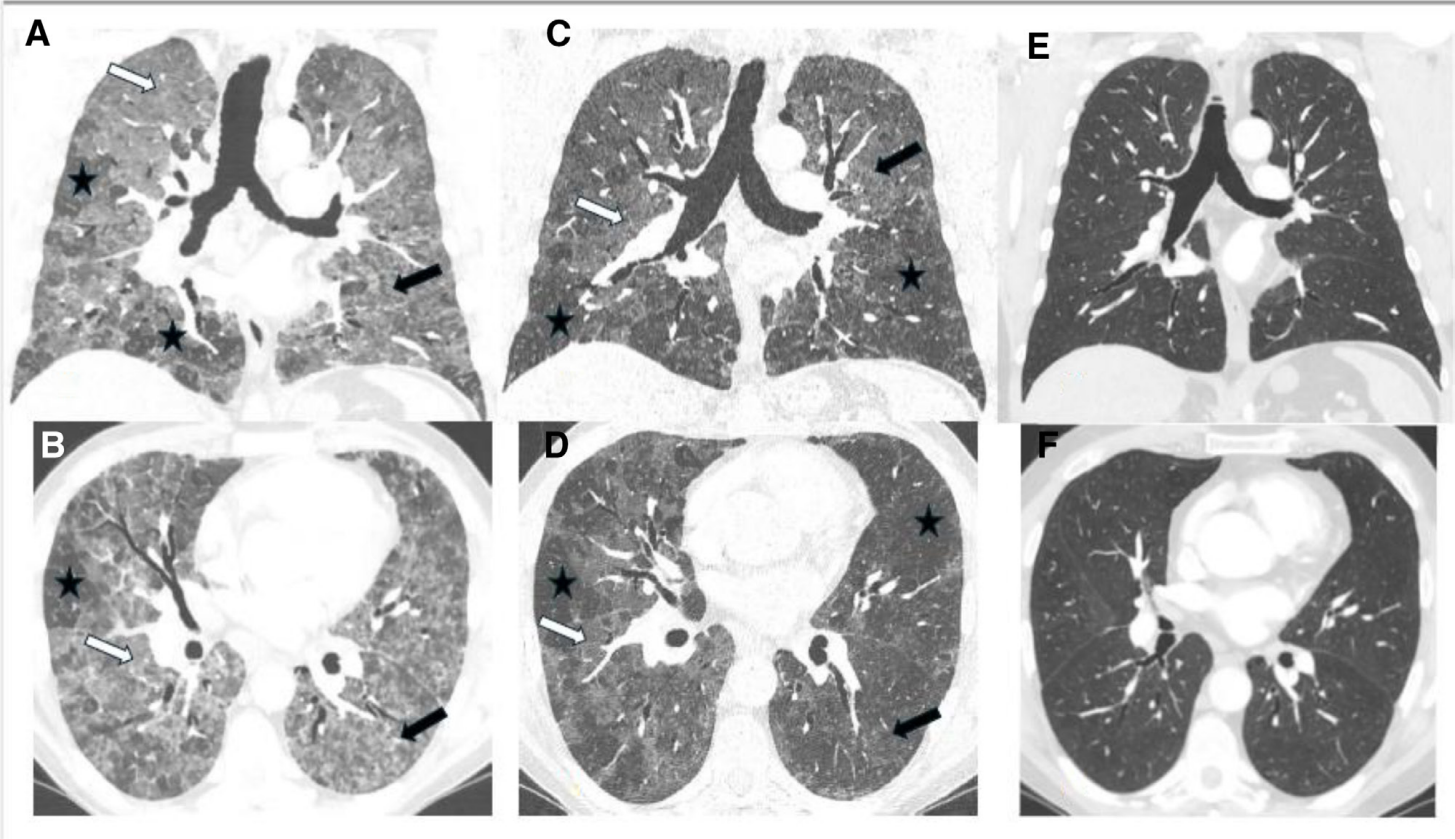
Patient 2 - HRCT scans coronal and axial planes. (A, B) baseline scans, (C, D) follow-up after 3 months, (E, F) follow-up after a year. (A, B) Lobular areas with poorly defined small centrilobular nodules (black arrows), ground glass opacities (white arrows) and with lobules of normal appearance suggestive of air trapping (asterisks). (C, D) Partial regression of the interstitial lesions; (E, F) Further (nearly complete) regression of lung opacities.

A 48-year-old male smoker (15 pack-years) was admitted to the pulmonary unit in July 2021 due to acute interstitial lung disease (ILD) with respiratory failure. He was a cattle breeder, feeding cattle with silage. Since April 2021, he reported worsening dyspnea, a cough with nonpurulent sputum, and episodes of low-grade fever, especially after working in the cowshed. Before admission to our hospital, he spent 2 weeks at a local facility, receiving broad-spectrum antibiotics and oxygen, but with no clinical improvement. On admission, he exhibited resting dyspnea, SpO_2_ of 83%, and bilateral inspiratory crackles throughout the lungs. Chest CT scan showed multiple, bilateral, ill-defined centrilobular nodules, some confluent, ground-glass opacities, and lobules with normal appearance suggestive of air trapping (Figure 2A, B).

Blood and sputum microbiologic cultures were negative. Nasopharyngeal smears for SARS–CoV–2 PCR were negative. HIV screening test was negative. Sputum *Pneumocystis jiroveci* PCR was negative. Cytomegalovirus PCR was negative. Epstein-Barr Virus antibodies (IgM and IgG) were negative.

Diagnosis of acute, non-fibrotic hypersensitivity pneumonitis was made based on exposure data, clinical presentation, and typical HRCT features.

Oxygen therapy at 7 L/min was administered through a face mask with reservoir, along with methylprednisolone 1 mg/kg/day (80 mg/day). By the third day of therapy, body temperature normalized, and oxygen needs decreased to 5 L/min. After 5 days, oxygen therapy was discontinued. The steroid treatment continued with prednisone 60 mg/day, with doses gradually reduced to 30 mg/day. Three months later, the patient was in good performance status and no longer needed oxygen support. HRCT showed significant, partial regression of lung attenuation (Figure 2C, D). The results of pulmonary function tests are shown in Table 1.

The patient still had contact with his cattle; however, he was using a face mask with a filter while working in the cowshed. It was decided to gradually lower the prednisone dose to 10 mg/day. In July 2022, a chest CT revealed nearly complete resolution of lung opacities (Figure 2E, F).

### 2.3. Case 3

**Figure 3.**
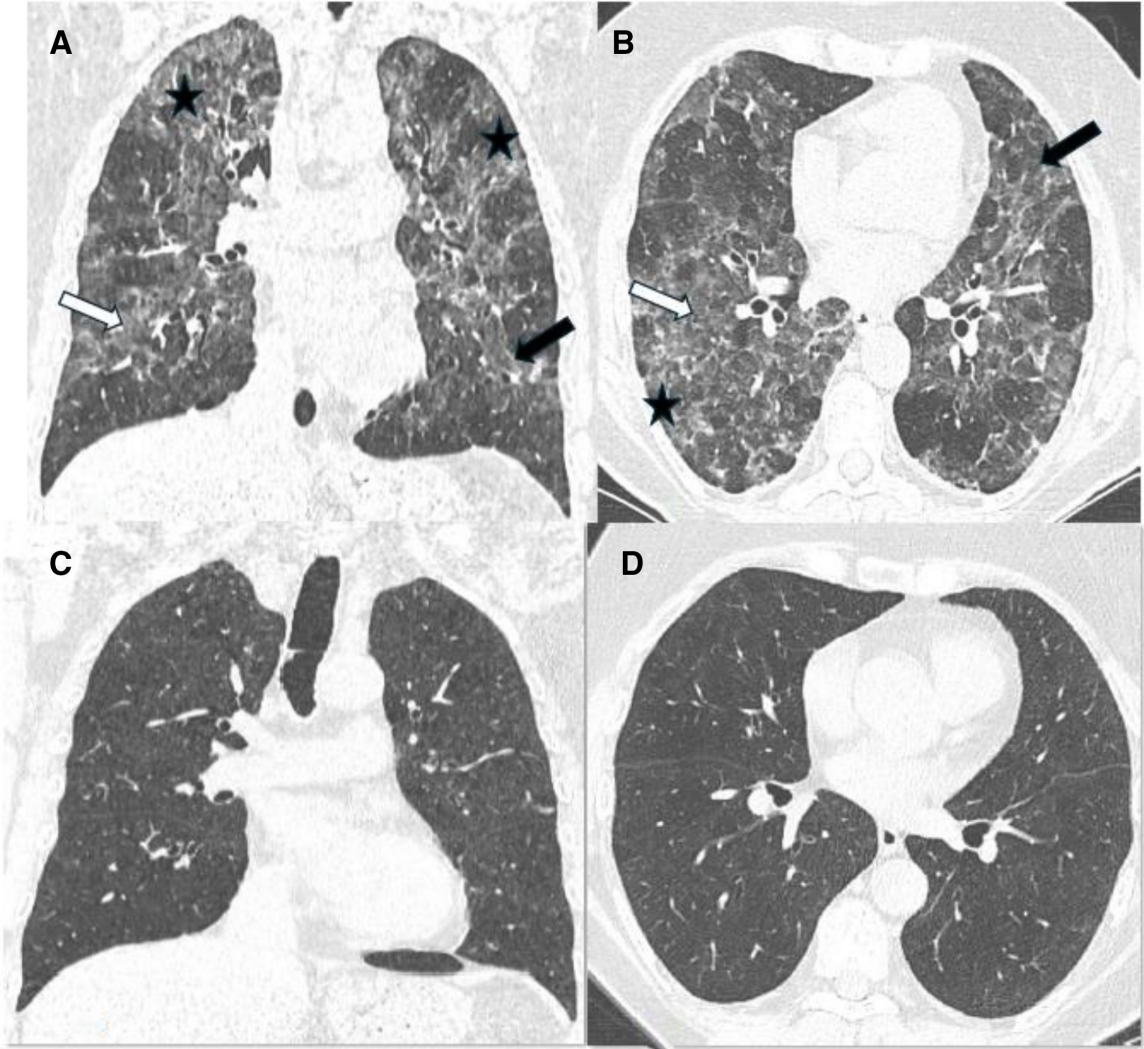
Patient 3 - HRCT scan, coronal and axial planes. (A, B) baseline scans: In the whole lungs bilaterally diffuse nodular consolidations (black arrows) and ground glass opacities (white arrows), with the reticulation pattern in peripheral lung regions with signs of lung fibrosis (asterisk). (C, D) follow-up scans: Complete regression of pulmonary infiltrates and no signs of lung fibrosis.

A 70-year-old woman, former smoker with 20 pack-years and diagnosed with Hashimoto disease treated with supplemental therapy, was referred to our clinic from the University Hospital in January 2019 due to suspicion of HP. She lived in her own house heated with wood, owned 2 cats, and used a feather duvet. Since September 2018, she has experienced a nonproductive cough, low-grade fever, and weight loss of 12 kg. On admission, she exhibited resting dyspnea, an SpO_2_ of 90%, and bilateral respiratory crackles over the lung bases. Chest CT showed bilateral, diffuse nodular consolidations and ground-glass opacities, with a reticulation pattern suggestive of early lung fibrosis. Additionally, a mosaic lung attenuation pattern (three-density pattern) was observed (Figure 3A,B).

BAL performed at the previous hospital showed 54% lymphocytes. Sputum cultures and *Legionella pneumophila* antigen in urine were negative. The early stage of fibrotic hypersensitivity pneumonitis was initially diagnosed. Oxygen at 4 L/min through a nasal catheter and prednisone at 30 mg/day were administered. After 7 days of treatment, oxygen therapy was discontinued. Prednisone was gradually tapered to 20 mg/day, and lung CT scans revealed nearly complete regression of pulmonary infiltrates with no signs of lung fibrosis (Figure 3C,D). Additionally, increases in lung volumes and a significant rise in TLco were observed (Table 1). Further tapering of prednisone was planned, but in March 2022, at a dose of 5 mg/day, she experienced clinical, radiological, and functional disease progression. She denied contact with animals and stopped using feather duvets, although she continued living in the same house. As a result, the steroid dose was temporarily increased to prednisolone 16 mg/day, then tapered to 8 mg/day. No further relapses occurred.

## 3. Discussion

Hypersensitivity pneumonitis is a granulomatous inflammatory lung disease that develops as a result of a pathological immunological reaction to the inciting antigen.^[14]^ Most patients report inhaling organic substances as the source of exposure. In our cases, various exposures have been noted: to hay straw (case 2), avian antigens (cases 1 and 3), and home-related factors (cases 1 and 3). Literature on this topic often describes exposure to avian proteins, not only in breeders but also among neighbors or those using feather duvets.^[12,15–17]^ An important cause of acute, non-fibrotic HP called summer-type HP is *Trichosporon asahii*.^[18]^ This exposure was described in Japanese patients living in wooden houses during humid summers.^[18]^ Summer-type HP presents as an acute disease with fever and malaise, a non-fHP pattern on HRCT, and a marked lymphocytic predominance in BAL.^[19]^ Two of our patients (cases 1 and 3) lived in village homes heated with wood and may have been exposed to similar microorganisms. An increasing number of HP cases due to bacterial or mold contamination of air conditioning devices or humidifiers has also been reported.^[15,16,20,21]^ Molds are present in 30–50% of energy-efficient buildings due to inadequate indoor air exchange rates.^[22]^ Additionally, exposure to mold antigens in foam pillows and mattresses has been documented.^[23]^ Recently, an acute form of non-fHP has been diagnosed in individuals who vape.^[24,25]^

The pathological inflammatory lesions in HP patients involve small bronchi and lung alveoli, leading to significant changes in gas exchange. Acute respiratory failure is rare but can be a life-threatening condition. This clinical course, which requires urgent medical interventions, has been observed in all of the presented patients.

Differential diagnosis of non-fHP with acute symptoms, such as fever and diffuse ground-glass opacities on HRCT, must include infectious causes of lung disease. In the era of the COVID-19 pandemic, SARS-CoV-2 pneumonia was considered the primary diagnosis, especially in patients requiring high-flow oxygen therapy. In 2 of our patients diagnosed at that time, SARS-CoV-2 infection was ruled out based on negative PCR tests from nasopharyngeal swabs. Infection with *Legionella pneumophila* was also excluded based on the negative urine antigen test results. The patients had no history of immunosuppression; nonetheless, *Pneumocystis jirovecii* pneumonia was excluded based on negative PCR results from sputum (cases 1 and 2) and bronchial washings (case 1).

Acute presentation of non-fHP may resemble organic dust toxic syndrome (ODTS).^[26,27]^ The differences and similarities between the 2 conditions are outlined in table 2.

**Table 2:** Clinical and radiological signs of disease in non-fibrotic hypersensitivity pneumonitis compared to organic dust toxic syndrome.

Parameter	Non-fHP	ODTS
Prior sensitization to organic antigens	present	not required
Inciting factors	Bacteria, fungi, animal proteins	Bacterial or fungal toxins present in organic dust
Specific IgG antibodies	May be present	Absent
HRCT	Typical pattern—combination of small airways disease and interstitial lung disease	No significant changes
BAL	Increased number of lymphocytes	Increased number of neutrophils
Hypoxemia	May be severe	Moderate
Fever, malaise, myalgia	May be present	Present in most cases

BAL = bronchoalveolar lavage, HRCT = high resolution computed tomography, non-fHP = non-fibrotic hypersensitivity pneumonitis, ODTS = organic dust toxic syndrome.

The diagnosis of non-fHP is based on characteristic HRCT patterns of changes, such as ill-defined centrilobular nodules, ground-glass opacities, and mosaic lung attenuation in a patient exposed to the inciting antigen.^[6,28]^ A typical non-fHP pattern is diagnosed if at least 1 sign of small airways disease (centrilobular nodules or air trapping) and 1 sign of interstitial lung disease (ground-glass opacities or mosaic lung attenuation), disseminated in axial and cranio-caudal projections, are present.^[6]^ Such radiologic features were found in all our patients. According to the recent American College of Chest Physicians (ACCP) recommendations, HP may be diagnosed without performing BAL in a patient with symptomatic lung disease caused by exposure to certain antigens and a typical HP pattern on HRCT.^[28]^ However, according to ATS guidelines, BAL lymphocytosis exceeding 30% is required, in addition to positive exposure history and typical HRCT pattern, to diagnose HP with high confidence.^[6]^ Severe hypoxemia present in all our patients was a contraindication for FOB with BAL. Nevertheless, in 1 patient (case 3), BAL was performed prior to admission to our department, and in another (case 1), BAL was performed after 10 days of prednisolone treatment. In both cases, lymphocytosis was documented, at 54% and 51%, respectively. In treatment-naïve non-fHP patients, BAL lymphocytosis may reach 40–70%,^[29]^ which is higher than in ILD due to collagen vascular diseases and sarcoidosis.^[30]^ Therefore, in our opinion, it is reasonable to perform BAL even in patients who have received a short course of steroid therapy, as the results may still be diagnostically valuable.

Treatment for non-fHP should focus on removing causative antigens from the patient environment. This approach requires thorough history-taking, considering all potential antigen sources. However, published studies show that up to 50% of HP patients do not identify the triggering antigen.^[15,16,31]^ To improve the chances of identifying exposure, questionnaires are recommended.^[6,28,32]^ Although many questionnaires have been used, none have been validated.^[15,20]^ In our patients, we used the Vasakova et al questionnaire^[20]^ to assess exposure history. All patients had exposure to organic antigens; however, only in case 2 was a correlation observed between exposure and new disease symptoms. Since exposure to organic antigens is common, it is important to ask whether symptoms worsen after exposure and improve after avoiding the antigen. According to Iijima et al, this approach helps prevent over-diagnosing HP based solely on positive exposure assessment results.^[33]^

The decision to start immunosuppressive therapy in non-fHP patients should be personalized, considering the severity of clinical signs and the amount of lung involvement seen on HRCT. All of our patients showed severe respiratory failure and extensive lung involvement on HRCT; therefore, the treatment choice was evident. We gave intravenous methylprednisolone at a dose of 0.5–1 mg/kg/day. Recent German, Japanese, and Korean guidelines recommend an initial dose equivalent to 0.5–1.0 mg of prednisone per kilogram of body weight for 4–6 weeks, followed by tapering over 2–3 months.^[13,25,34]^ Pulse steroid therapy (methylprednisolone 0.5–1 g/day for 3 days) can be used in patients with severe hypoxemia.^[13]^ In our cases, the clinical response to treatment was immediate, with respiratory failure improving within a few days. However, lung abnormalities seen on HRCT lasted much longer, even up to 7 months in case 2. Therefore, in our opinion, extended oral steroid therapy may sometimes be needed in non-fHP patients with extensive lung involvement to achieve the best possible respiratory recovery.

In case 3, extensive ground glass opacities and centrilobular nodules seen on HRCT coexisted with discrete reticulation. This HRCT pattern is difficult to classify as either non-fHP or fHP. The discussion about the radiologic definition of fHP is ongoing. According to ACCP guidelines, reticulation as a single HRCT sign is not enough to diagnose fHP unless other signs of lung distortion, such as bronchiectasis or decreased lung volume, are present.^[28]^ On the other hand, in some patients with extensive ground glass opacities, the presence of reticulation may be underestimated. Since a complete radiologic response to treatment was achieved in case 3, the disease was ultimately classified as non-fHP.

## 4. Summary

Non-fibrotic HP may rarely present as febrile lung disease, with extensive lung involvement causing acute respiratory failure. In such patients, it is important to rule out respiratory infection, especially during the SARS-CoV-2 pandemic. The presence of ill-defined pulmonary nodules and ground-glass opacities, along with a mosaic lung attenuation pattern on HRCT, is highly suggestive of non-fHP. In these cases, it is crucial to inquire about all potential exposures: environmental, home-related, and those related to work. In patients with severe hypoxemia, the diagnosis of non-fHP may be based solely on positive exposure history and characteristic HRCT findings. However, BAL performed after a few days of therapy can still provide diagnostic value. Resolution of respiratory insufficiency is usually observed within days of treatment with intravenous prednisolone at 0.5–1 mg/kg, though lung opacities may persist for several months. Therefore, prolonging steroid therapy in such patients is reasonable to prevent lung fibrosis. If exposure is unknown or not eliminated, long-term therapy with the lowest effective steroid dose should be considered.

## Acknowledgments

We acknowledge all patients who consented to use their data in this publication.

## Author contributions

**Conceptualization:** Katarzyna B. Lewandowska, Monika Szturmowicz.

**Project administration:** Katarzyna B. Lewandowska, Monika Szturmowicz.

**Software:** Katarzyna B. Lewandowska.

**Writing – original draft:** Katarzyna B. Lewandowska, Iwona Bartoszuk, Małgorzata E. Jędrych, Kamila Deutsch, Monika Szturmowicz.

**Formal analysis:** Lucyna Opoka.

**Visualization:** Lucyna Opoka, Lilia Jakubowska.

**Data curation:** Iwona Bartoszuk, Małgorzata E. Jędrych, Piotr Radwan-Rohrenschef, Kamila Deutsch.

**Investigation:** Piotr Radwan-Rohrenschef, Adriana Roży, Lilia Jakubowska.

**Writing – review & editing:** Piotr Radwan-Rohrenschef, Adriana Roży, Lilia Jakubowska, Witold Z. Tomkowski, Małgorzata Sobiecka.

**Supervision:** Witold Z. Tomkowski, Małgorzata Sobiecka, Monika Szturmowicz.
